# Strategic Placement of Pores to Modulate Toughness in Hydroxyapatite Bone Scaffolds Fixation

**DOI:** 10.3390/biomimetics11070479

**Published:** 2026-07-09

**Authors:** Hajar Souhail, Luca D’Andrea, Anna De Cet, Davide Ruffoni, Pasquale Vena

**Affiliations:** 1 Laboratory of Mechanics of Biological and Bioinspired Materials (MBBM), Department of Aerospace and Mechanical Engineering, University of Liège, Allée de la Découverte 9, 4000 Liège, Belgium; hsouhail@uliege.be; 2 Laboratory of Biological Structure Mechanics (LaBS), Department of Chemistry, Materials and Chemical Engineering “Giulio Natta”, Politecnico di Milano, Piazza Leonardo da Vinci 32, 20133 Milan, Italy; luca.dandrea@polimi.it (L.D.); anna.decet@polimi.it (A.D.C.); pasquale.vena@polimi.it (P.V.); 3 Department of Biomechanics, Faculty of Medicine, Karl Landsteiner University, Dr. Karl-Dorrek-Straße 30, 3500 Krems, Austria

**Keywords:** bone, bio-inspiration, fixation, ceramic scaffold, fracture

## Abstract

Hydroxyapatite-based ceramic scaffolds are attractive for bone tissue engineering but are prone to brittle fracture during screw fixation. This study addresses the challenge of tuning local toughness in the peri-screw region of brittle ceramic scaffolds. Inspired by the presence of voids in many load-bearing biological materials, we explored how micro-pores interact with cracks to modulate fracture toughness. A circular scaffold is considered, featuring a central hole to accommodate the screw and a notch to trigger fracture. The scaffold was analysed under radial compression, using finite element simulations based on a phase field formulation for brittle fracture. The influence of notch size on crack propagation was first investigated and compared with the behavior of a plate under mode I failure. Subsequently, the influence of micro-pore shape and spatial arrangement on strength and toughness was examined. Results showed that the circular scaffold attenuates notch sensitivity when compared to the plate-like scaffold, due to the interplay between circumferential tension and radial compression. Circular pores increased energy dissipation through crack deflection and they decreased the strength due to stress concentration. Elongated pores preserved strength by mitigating these stress peaks. These findings identify key geometric parameters for optimizing the mechanical reliability of ceramic scaffolds requiring screw fixation.

## 1. Introduction

**Figure 1:**
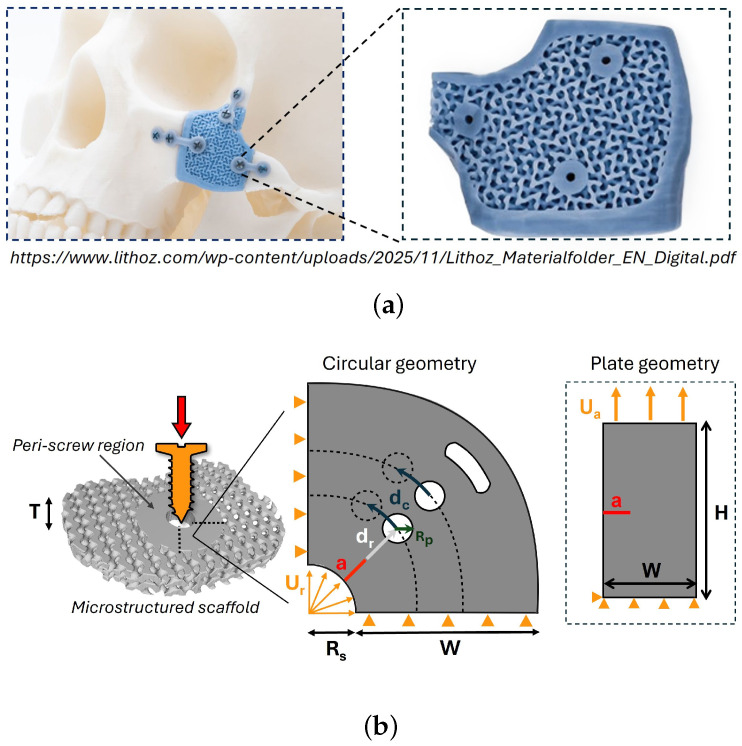
(**a**) Example of patient-specific porous hydroxyapatite implant used for maxillofacial reconstruction.The implant is custom-shaped to match the bone defect and secured to the host bone using fixation screw. Figure adapted from Lithoz.com with permission; (**b**) From left to right: 3D scheme of a scaffold, highlighting the circular peri-screw region of interest surrounding the central hole for screw fixation, as distinct from the surrounding microstructured scaffold; Simplified 2D model used for phase-field fracture simulations, including a hole radius $R_{s}$, a width *W*, a sharp notch of size *a* (in red) and pores with different shapes (circular with radius $R_{p}$ or elongated) placed at different locations. Grey and blue arrows with respective labels $d_{r}$ and $d_{c}$ indicate the arrangement of pores. Orange arrows indicate the radial displacement ($U_{r}$) applied to simulate screw expansion, while orange triangles represent the symmetry constraints (circumferential displacement $U_{\theta }=0$) along the boundaries; Plate-like geometry of width W and height H, enclosed in a dashed rectangle to indicate that it serves as a reference geometry, including a crack of length *a* in red and subjected to an axial displacement $U_{a}$ (orange arrows), with symmetry constraints along the lower boundary (orange triangles).

Bone grafting is used to repair or replace bone when natural healing is insufficient, such as in non-union fractures, large bone defects caused by trauma or tumor removal, spinal fusion procedures, and revision joint surgeries [1,2,3]. It is also widely applied in neurosurgery for cranial defects as well as in maxillofacial surgery and dentistry to restore bone volume for jaw reconstruction and dental implants [4,5] (Figure 1a). The gold standard for a bone graft is autogenous grafting, where bone is taken from the patient’s own body and transplanted to another site to promote bone healing or regeneration. This approach comes with some drawbacks including a second surgical procedure and the limited amount of bone available [6,7].

As a potential substitute for autogenous free bone grafting, hydroxyapatite (HA)-based implants have attracted considerable interest in recent years [8]. Synthetic HA exhibits excellent biocompatibility and strong osteoconductive properties, promoting effective osseointegration [9,10]. Because of these favorable properties, porous HA structures have been developed to further enhance biological integration. The open-pore architecture supports both bone conduction and mechanical stability, making them suitable for clinical use in orthopedics and maxillo-facial surgery [11,12]. The use of advanced 3D printing technologies offers promising potential for expanding HA clinical applications. Several additive manufacturing (AM) techniques have been explored for the fabrication of HA-based scaffolds. Extrusion-based methods such as robocasting, while widely adopted for their simplicity and compatibility with self-setting HA inks, are limited by relatively low resolution and restricted geometric complexity. Powder bed fusion approaches, including selective laser sintering (SLS), enable support-free fabrication but suffer from limited densification and residual porosity. Binder jetting deposits a liquid binding agent onto HA powder layers allowing rapid production of complex geometries, although the resulting parts typically require demanding post-processing steps to achieve adequate densification [13]. Among these techniques, vat photopolymerization, encompassing stereolithography and digital light processing, stands out for its superior dimensional accuracy, enabling high-resolution control over porosity, pore size, and wall thickness and allowing for the fabrication of highly customized implants tailored to patient needs [11]. However, challenges remain, including the intrinsic brittleness of ceramic materials [14], which limits the load bearing ability of the implant and its potential fixation to the host bone. In clinical practice, particularly in maxillofacial and dental applications, HA-based scaffolds are commonly stabilized using fixation screws to ensure primary stability during healing, while osseointegration progressively secures long-term fixation. This is typically achieved through dedicated screw-holes integrated into the scaffold design [4,5]. Investigating the mechanical interaction between synthetic HA scaffolds and titanium screws has highlighted that failure due to screw insertion and loading is a critical factor limiting the mechanical reliability of ceramic implants [15]. Therefore, particular attention must be paid to the fixation strategies when ceramic scaffolds are secured with screws. With the advent of additive manufacturing, it is now possible to directly incorporate screw-accommodating holes within the scaffold, thereby avoiding drilling-related damage. Specifically the hole diameter can be designed to be either slightly larger or slightly smaller than the screw thread diameter. In the first case, fixation is achieved primarily through compressive axial stresses (i.e., along the screw axis) exerted by the screw head on the scaffold surface, while the screw threads engage mainly with the underlying bone tissue. In the second case, the screw is directly threaded into the ceramic scaffold, inducing radial compressive stresses (i.e., perpendicular to the screw axis) at the hole boundary that generate circumferential tensile stresses in the surrounding material–a loading condition that is particularly critical for brittle ceramics, which are much weaker in tension than in compression [16,17]. In both scenarios, failure depends on the fracture toughness of the ceramic scaffold, the applied load and the mismatch between hole and screw diameters. Understanding these failure mechanisms is therefore critical to prevent scaffold fracture at the time of screw insertion during surgery. However, improving the intrinsic brittleness of ceramics is challenging and there is also a fundamental trade-off between strength and toughness: enhancing one typically comes at the expense of the other [18]. This difficulty is exacerbated by the negligible plasticity of ceramics: once a crack forms, it tends to propagate uncontrollably [19]. Beyond compositional modifications, microstructural variations can also be used to explore the balance between strength and toughness. For example, computer simulations of brittle, porous 3D-printed scaffolds under uniaxial compression suggest that optimized microstructural design can increase toughness, while leading to a reduction in strength [20].

Interestingly, biological materials can combine relatively high strength with high toughness, through sophisticated hierarchical structures built with a limited set of chemical elements [21]. Bone, for instance, exhibits extraordinary fracture resistance. This is enabled by a multiscale structure, where properties can be tuned at various structural levels [22,23]. At the nanoscale, the britte mineral particles are glued by a tough collagenous matrix, which flows plastically above a critical stress, thereby protecting the particles [24]. The nanoscale dimensions of the particles, together with the compressive stresses induced by the collagen matrix on the particles during mineralization, are additional factors enhancing nanoscale strength [25,26]. At the fibrillar and sub-fibrillar scales, the collagen-matrix provides toughening by fibril sliding and molecular uncoiling [27]. At larger structural levels, the lamellar organization is a crucial contributor to bone toughness, providing energy dissipation by crack deviation and twisting through the lamellae [28]. Cortical bone is also full of pores of different dimensions to host blood vessels, cells and cell processes. Large pores are shielded by specific structures called osteons, where a central hole is protected by lamellar bone and bordered by a specialized interface, the cement line, which is more mineralized [29], stiffer and harder than the osteons [30]. Cracks are often deflected around the osteons, along low-energy paths, thereby dissipating energy and delaying catastrophic failure [31]. Small pores such as cell lacunae can have a dual effect: they can both act as stress concentrators, thereby triggering crack formation, but they can also slow down crack propagation by deviating and blunting the crack [32]. The crucial role of pores (voids) on fracture behavior was also highlighted in other biological material, such as the chitin-based exoskeleton of mantis shrimps [33,34], which is built with heavily mineralized components but shows remarkable damage tolerance [35,36,37].

There is huge interest to improve the toughness of engineering ceramics and replicating some of the design principles found in biological materials is a promising route [37,38,39,40]. Following a bio-inspired approach, it was possible to enhance the toughness of brittle glass up to 200 times, by engraving laser-generated holes in the glass to trap and guide crack into toughening configurations [41]. The introduction of controlled porosity or weak interfaces, into an otherwise monolithic material, can therefore promote crack deflection and increase the energy required for fracture propagation, thereby enhancing the fracture toughness. In particular, in the peri-screw region, which is a mechanically critical zone, it is relevant to investigate whether a controlled porous architecture can be retained rather than assuming a fully dense design. Building on these concepts, the present study investigates how the placement of pores with different shape and at different positions in this region can modulate the toughness with respect to the dense configuration. Focusing on screw fixation into a pre-drilled hole slightly smaller than the screw shaft, we used computer simulations to study crack patterns, strength and toughness.

## 2. Materials and Methods

We used a series of finite element (FE) simulations to investigate the influence of pore position and shape on fracture behavior of circular-shaped scaffolds, representative of the region surrounding a screw in 3D-printed bioceramic implants (Figure 1b). It is assumed that the region close to the central fixation hole is made of bulk HA, whereas the microstructured scaffold is placed far from the fixation screw and therefore not reported in the FE model. In other words, the scaffold comprises two distinct microstructures: one located away from the screw, usually optimized to promote bone ingrowth while ensuring sufficient biomechanical stability, and another located in the peri-screw region, specifically tailored to accommodate the challenging mechanical environment caused by the screw as well as to still enable bone ingrowth. The circular scaffold was represented through a simplified two-dimensional (2D) model, under plane stress conditions with unit out-of-plane thickness T (Figure 1b). Exploiting circular symmetry to contain computational costs, only a quarter of the scaffold was considered, having a width *W* of 8 mm, defined as the difference between the outer radius (9 mm) and the inner hole radius $R_{s}$ of 1 mm (corresponding to 12.5% of scaffold width W), representative of the pre-drilled hole size compatible with fixation screws typically used in porous hydroxyapatite implants [15]. A sharp notch, with size *a*, was introduced at the midpoint of the inner surface of the hole to act as fracture initiation site (Figure 1b). Screw insertion was modeled by applying a uniform radial displacement $U_{r}$ of 0.004 mm (corresponding to 0.05% of scaffold width) along the inner boundary of the scaffold, replicating the expansion effect induced by screw placement. To enforce symmetry, circumferential displacements $U_{\theta }$ were constrained along the lower and right boundaries, which represent the axes of symmetry. Fracture simulations were performed using a phase-field formulation for brittle materials [42], implemented in *Abaqus/CAE* through a user-defined material subroutine (UMAT) [43,44]. Within this implementation, crack evolution is governed by a scalar damage variable ϕ, ranging from 0 (intact) to 1 (fully damaged), which evolves continuously in the domain based on a variational principle linking the strain energy density Ψ and the energy release rate $G_{c}$. Specifically, Ψ is obtained from the elastic strain field computed at each increment, acting as the mechanical driving force for the crack growth. The governing equation of the phase-field damage variable is expressed as:

(1) $$\begin{array}{c} \nabla^{2}\phi =\frac{\phi }{\ell^{2}}-\frac{2(1-\phi )}{G_{c}\ell }\Psi \end{array}$$

where *ℓ* represents the phase field length scale, which governs the size of the damaged region. To account for the typical asymmetry between tensile and compressive strengths in brittle ceramics, a Drucker–Prager-based energy decomposition was applied [43]. Specifically, the tensile strength was set to 100 MPa [9], while the compressive strength was assumed to be ten times higher (1000 MPa), consistent with the 10:1 ratio typical for ceramic materials as reported by Munz and Fett [16]. The material elastic properties used for the simulations were: *E* = 100 GPa and ν = 0.3 [9]; the fracture parameter $G_{c}$ was set to $10\;{J/m}^{2}$, computed as $G_{c}=K_{Ic}^{2}/E$, with $K_{Ic}$ = 1 MPa$\sqrt{m}$ [45]. To assess the robustness of the numerical framework with respect to this parameter, a sensitivity analysis was performed considering $G_{c}$ values of 20, 5, 2, 1, and 0.5 J/m^2^, i.e., spanning 0.05–2 times the reference value (10 J/m^2^). The influence of $G_{c}$ variations was evaluated in terms of the maximum principal stress along the crack propagation path, used as a representative output due to its direct relation with the material’s intrinsic fracture resistance. To accurately resolve the damage field, the domain was discretized using thermally coupled 4-node plane stress elements. These elements were employed to exploit the analogy between the phase-field evolution equation and the heat transfer problem, where the nodal temperature is reinterpreted as the scalar damage variable ϕ. The smallest mesh element size was set to 1% of the scaffold width, ensuring it remained at least two times smaller than the phase-field length scale (*ℓ*) to properly capture the damage gradient. The main simulation parameters are summarized in Table 1.

The reaction force versus the imposed displacement data were obtained from the simulations and used to compute the nominal stress-strain curves. Strength was defined as the maximum stress and toughness was approximated as the area under the stress-strain curve. Results were visualized in terms damage variable, a surrogate descriptor of crack propagation. The following three main aspects were systematically studied:

- Notch sensitivity: The notch size *a* was varied from 5% to 50% of the scaffold width *W*, considering a/W=0.05,0.10,0.15,0.20, and 0.50. Although the circular scaffold was loaded radially, the resulting circumferential stress was tensile and oriented perpendicular to the crack plane, analogously to a standard mode I fracture configuration. A plate-like geoemtry of the same width W and height H≈2W was therefore introduced as a numerical reference case with a comparable tensile stress state (Figure 1b), allowing the notch sensitivity of the circular scaffold to be benchmarked against a simpler and more interpretable geometry. For the circular scaffold, the radial stress was obtained based on the reaction forces associated with the imposed radial displacement at the inner boundary divided by the corresponding resistant surface. It should be noted that the circumferential stress, oriented perpendicular to the crack plane, is linearly proportional to the radial stress, according to the theory of thick-walled cylinders under pressure. The circumferential strain was derived from the radial displacement at the inner boundary as $\varepsilon_{\theta }=U_{r}/R_{s}$, which directly reflects the crack-opening deformation mode. In the plate-like scaffold, the mechanical response was described in terms of tensile stress and strain, derived from the reaction force and axial displacement at the loaded edge $U_{a}$, with the tensile strain given by $\varepsilon =U_{a}/H$. To enable a direct comparison of notch sensitivity across different notch sizes and between the two geometries, all stress values were normalized with respect to the maximum stress obtained for the smallest-notch configuration (i.e., a/W = 0.05).
  In addition, to verify whether the simulations behave consistently with Griffith’s law, the normalized strength, as a function of a/W, was compared against the analytical prediction based on the brittle fracture criterion. In this framework, the fracture stress decreases with crack size as $\sigma \propto 1/\sqrt{\pi a}$, corrected by the geometry-dependent shape factor $f_{S}\left(a/W\right)$ to account for finite-width effects [46]. The analytical model takes the crack size *a* and the plate width *W* as inputs and returns the normalized fracture stress as output, according to the following equation:
  (2) $$\sigma \approx \frac{1}{\sqrt{\pi a}\cdot f_{S}\left(\frac{a}{W}\right)}=\frac{1}{\sqrt{\pi a}}{\left[\sqrt{\frac{2W}{\pi a}\tan\frac{\pi a}{2W}}\cdot \frac{0.752+2.02\frac{a}{W}+0.37{\left(1-\sin\frac{\pi a}{2W}\right)}^{3}}{\cos\frac{\pi a}{2W}}\right]}^{-1}$$
- Pore position: (*I*) A single pore of radius $R_{p}=0.5$ mm (corresponding to 6.25% of scaffold width) was placed in front of the notch. The chosen pore dimension represents a compromise between mechanical performance (large cavities would weaken the scaffold) and manufacturability (removal of uncured material). This pore size falls within the range typically reported for microstructured hydroxyapatite scaffolds produced by additive manufacturing [13,47]. The radial notch-pore distance ($d_{r}$ in Figure 1b), defined as the distance between the notch tip and the center of the pore along the radial direction, was varied from 2 $R_{p}$ to 3.5 $R_{p}$. (*II*) The pore (placed at a constant $d_{r}$ = 2 $R_{p}$) was then moved along a circumferential trajectory, with the circumferential notch-pore distance ($d_{c}$ in Figure 1b) defined as the angular offset of the pore from the notch tip along the same circular arc. (*III*) Two more pores of identical radius were added, so that all three pores were arranged radially and equally spaced in front of the notch. Starting from this configuration, the middle pore was shifted along the circumferential direction by gradually increasing $d_{c}$. In all configurations, pores are modelled as non-interconnected features, to isolate the effect of their spatial arrangement on fracture behavior. To facilitate comparison among different scenarios, stresses were normalized with respect to the maximum stress of the corresponding baseline configurations (i.e., no pore for *I*, one pore at $d_{c}$ = 0 for *II*, and three aligned pores for *III*).
- Pore shape: To investigate the effect of pore shape on fracture behavior, the circular pores were replaced by circumferentially elongated pores of equal area (Figure 1b). Specifically, the circumferential elongation was chosen to reflect the directional stress distribution induced by the geometry of the region surrounding the central fixation hole. Three elongated pores were considered: initially aligned radially in front of the notch (analogously to configuration *III* of the pore position study), and then with the middle pore progressively shifted along the circumferential direction by increasing $d_{c}$. Results were compared with the circular pore configurations placed at the same locations.

Post-processing of the FE simulations was conducted using Abaqus to extract mechanical data, including nodal displacement and 2D damage patterns. MATLAB (MathWorks, R2024a) and Excel (Microsoft, 365) were used for data handling and visualization, respectively.

## 3. Results

### 3.1. Notch Sensitivity

**Figure 2:**
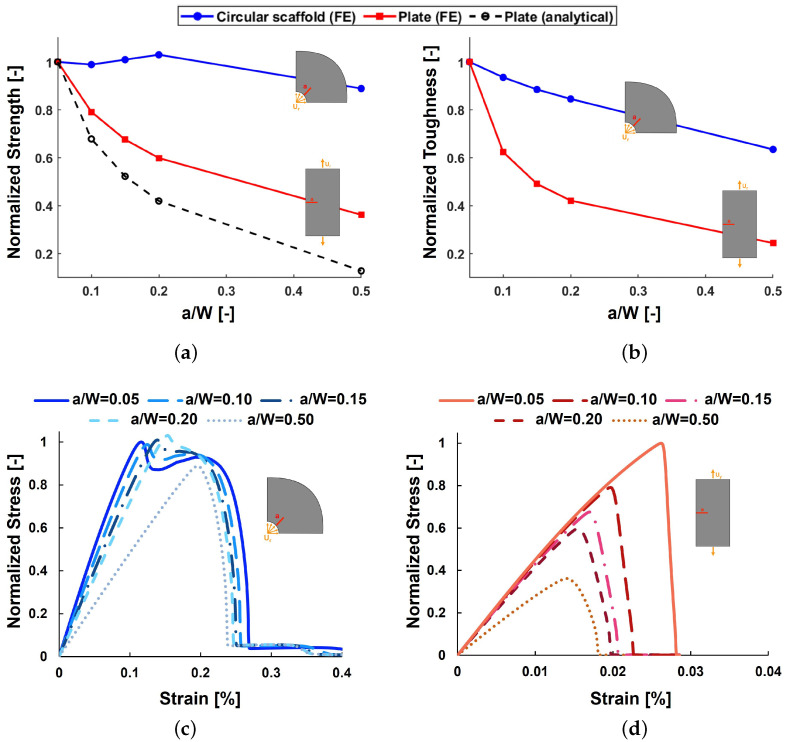
Comparison between the mechanical response of a circular scaffold and a plate under increasing notch size a/W. Normalized strength and toughness as a function of a/W for the circular (**a**) and the plate (**b**) scaffold; Normalized stress–strain responses for varying a/W values in the circular (**c**) and the plate (**d**) scaffold, respectively.

Figure 2a shows the normalized strength of the circular- and plate-like scaffolds as a function of the notch size a/W. In the plate, the strength decreases monotonically with increasing notch size, following the trend predicted by Equation (2), with the analytical prediction providing a lower bound to the numerical results. A similar decrease is observed for the normalized toughness (Figure 2b). In contrast, the circular scaffold exhibits a markedly different response. The normalized notch-opening strength remains nearly constant over the investigated range of notch sizes. The global mechanical response underlying this behavior is illustrated by the stress–strain curves reported in Figure 2c. For the circular scaffold, the peak radial stress, associated with the onset of crack initiation, exhibits only minor variations across different notch sizes. Up to a notch size a/W = 0.2, all curves were fairly similar: following the peak stress, a gradual decay and a finite transition region are observed before complete failure. For a larger notch size (a/W = 0.5) the curve suggests a more brittle behaviour. In the plate, increasing notch size leads to a pronounced reduction in strength, and for all notch configurations, the peak stress is followed by a sudden drop (Figure 2d).

### 3.2. Pore Position

#### 3.2.1. Radial Notch-Pore Distance

**Figure 3:**
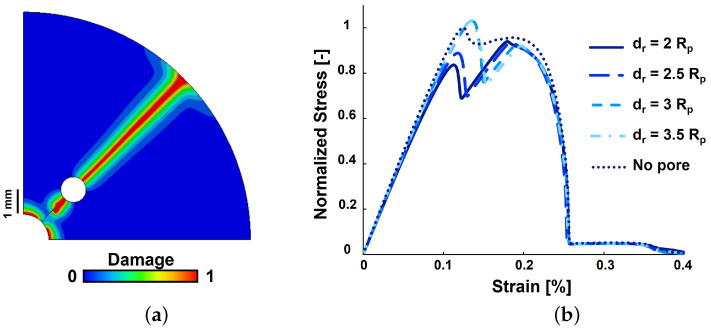
Effect of the radial notch–pore distance $d_{r}$ on fracture behavior. (**a**) Damage pattern for the representative case $d_{r}$ = $2R_{p}$: in red fully damaged elements; (**b**) Normalized stress–strain for increasing values of distance $d_{r}$.

The influence of the radial distance between the notch and one single pore ($d_{r}$ in Figure 1b) was investigated by varying $d_{r}$ between $2R_{p}$ and $3.5R_{p}$, while keeping the notch size fixed at a/W=0.10. Figure 3a shows the damage distribution for a representative case ($d_{r}$ = $2R_{p}$). For all values of $d_{r}$, similar crack paths were observed, with fracture initiating at the notch and propagating straight into the pore. When exiting the pore, the crack proceeded along the same direction. The corresponding normalized stress–strain curves are shown in Figure 3b, where the stress is normalized with respect to the maximum stress of configuration without the pore. For all pore locations, the response exhibits a characteristic double-peak profile. When the pore is positioned closer to the notch (i.e., $d_{r}$ = $2R_{p}$), both peaks occur at lower normalized stress levels compared to the no-pore configuration, with the second peak remaining higher than the first. As the distance $d_{r}$ increases (i.e., $d_{r}$ = $3R_{p}$), the first peak increases in magnitude, approaching the no-pore response, while the second peak becomes less pronounced.

#### 3.2.2. Circumferential Notch–Pore Distance

**Figure 4:**
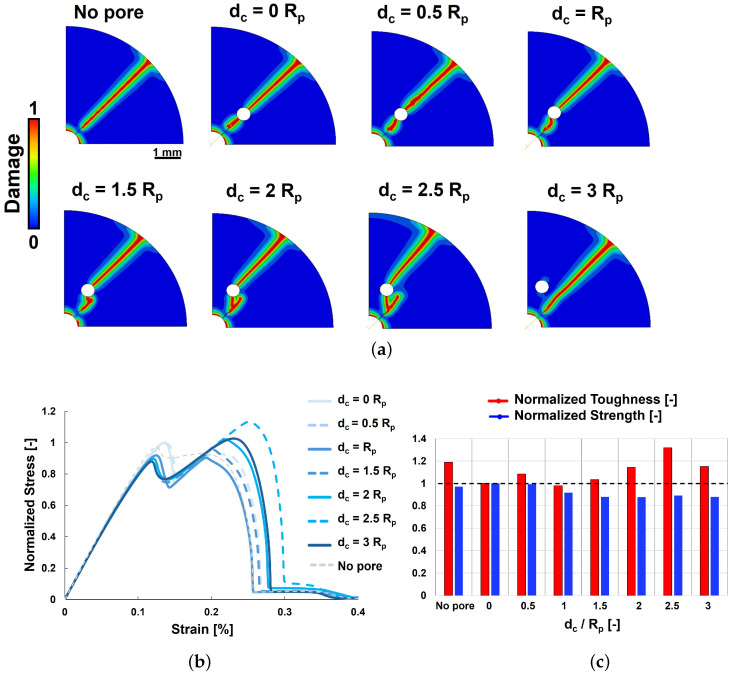
Effect of the circumferential notch–pore distance $d_{c}$ on fracture behavior. (**a**) Damage pattern for the no-pore configuration and at different values of $d_{c}$: in red fully damaged elements; (**b**) Normalized stress–strain curves for the no-pore configuration and at increasing values of distance $d_{c}$; (**c**) Normalized strength–toughness plots at increasing values of distance $d_{c}/R_{p}$, including the no-pore case; the dashed line indicates the value equal to 1.

The effect of varying the circumferential offset between the notch and one single pore ($d_{c}$ in Figure 1b), was investigated over a range from 0 to $3R_{p}$, again at notch size a/W=0.10. As shown in Figure 4a, the cracks originate from the notch but follow different paths depending on the value of $d_{c}$. For offsets up to $2.5R_{p}$, the crack was attracted and entered into the pore, exhibiting noticeable circumferential deviation. When exiting the pore of radius $R_{p}$, the crack followed a radial path. In contrast, for higher values of $d_{c}$, the crack was no longer attracted by this pore and propagated radially without deviation. The corresponding mechanical response is shown in Figure 4b, where the stress is normalized with respect to the first peak stress obtained for the reference configuration ($d_{c}=0R_{p}$). As the offset increases, the peak stress tends to decrease, resulting in a progressive reduction in strength. The area of the curves reflect the non-monotonic behavior of toughness: it increases initially (local maximum at $d_{c}\approx 0.5R_{p}$) and then rises again to a global maximum at $d_{c}\approx 2.5R_{p}$, as summarized in Figure 4c. The no-pore configuration exhibits both higher toughness and strength compared to most circular pore configurations.

#### 3.2.3. Circumferential Inter-Pore Distance

**Figure 5:**
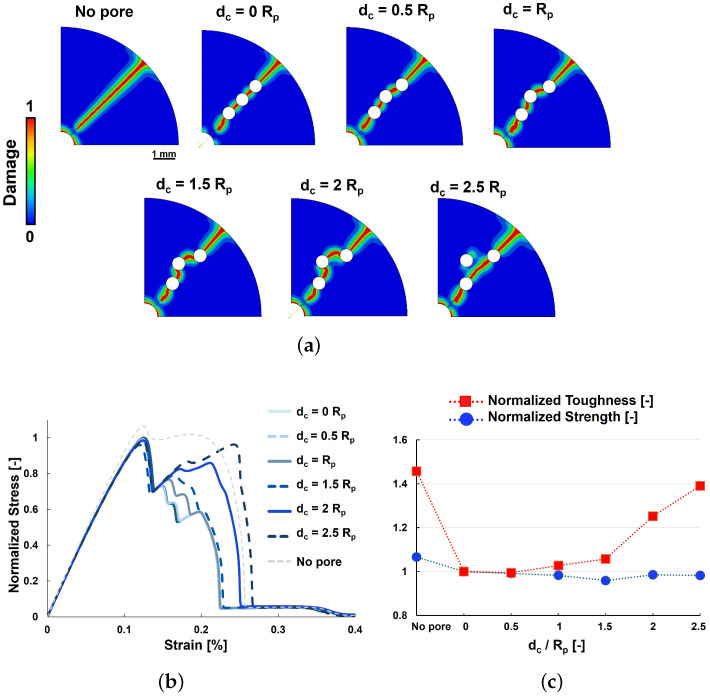
Effect of the inter-pore distance $d_{c}$ on fracture behavior. (**a**) Damage pattern for the no-pore configuration and at different values of $d_{c}$: in red fully damaged elements; (**b**) Normalized stress–strain curves for the no-pore case and at increasing values of distance $d_{c}$; (**c**) Strength–toughness plot for no-pore reference and for varying values of $d_{c}$.

The influence of pore position was further investigated using a three-pore configuration, where the parameter $d_{c}$ is defined as the circumferential distance between the intermediate pore and the two adjacent ones along the same radial level. As shown in Figure 5a, for small values of $d_{c}$, the crack follows a nearly radial trajectory, remaining aligned with the pores. As $d_{c}$ increases, the attractive influence of the intermediate pore becomes evident, as the crack path gradually deviates more noticeably toward it. This crack-attraction mechanism eventually leads to the crack bypassing the pore entirely at $d_{c}=2.5R_{p}$, indicating a transition in the fracture pattern. The mechanical response, reported in Figure 5b, shows that the normalized stress–strain curves change with increasing $d_{c}$. As the inter-pore distance grows, the peak stress progressively decreases, while the area under the curve increases, indicating a corresponding rise in toughness. This behavior is summarized in the normalized strength–toughness plot in Figure 5c, where toughness increases with $d_{c}$, whereas strength remains practically constant. These trends are consistent with the longer and more tortuous crack paths observed for larger pore spacing, which enhance energy dissipation but reduce structural integrity. This behavior is quantitatively reflected in the crack path length, which increases from 5.46 mm at $d_{c}=0R_{p}$ to 6.49 mm at $d_{c}=2.5R_{p}$, with intermediate values of 5.53, 5.66, 6.09, and 6.45 mm at $d_{c}=0.5R_{p}$, $1R_{p}$, $1.5R_{p}$, and $2R_{p}$, respectively. Notably, the no-pore configuration exhibits the longest crack path length (8.40 mm) and the highest toughness, as reported in Figure 5c.

### 3.3. Pore Shape

**Figure 6:**
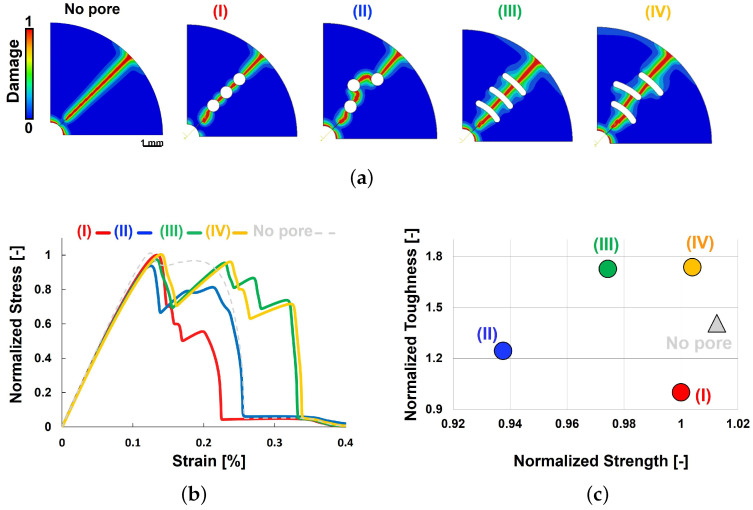
Effect of the pore shape on fracture behavior. (**a**) Damage pattern in different scenarios: no-pore configuration, radially aligned circular (I) and elongated (III) pores; staggered circular (II) and elongated (IV) pores; (**b**) Normalized stress–strain curves for the five different scenarios; (**c**) Normalized strength–toughness plot for the five different scenarios.

Figure 6 compares four pore designs (I–IV) in terms of damage evolution (a), stress–strain response (b), and strength–toughness trade-off (c). In the damage maps (Figure 6a), design (I) features radially aligned circular pores, resulting in a straight crack path. Design (II) introduces a circumferential offset central pore, which redirects the crack along a more curved trajectory. Designs (III) and (IV) replace the circular pores with circumferentially elongated ones of equal area, radially aligned in design (III) and with one pore staggered circumferentially in design (IV). Unlike circular pores, where the crack resumes propagation from a surface characterized by the pore’s radius of curvature, elongated pores force the crack to restart from a comparatively flatter, less curved surface. The mechanical response, shown in Figure 6b, reveals that designs (III) and (IV) exhibit delayed failure and higher load-bearing capacity compared to (I) and (II). The corresponding normalized strength–toughness diagram (Figure 6c) confirms this trend, with designs (III) and (IV) occupying the upper right quadrant, indicating improved energy dissipation without compromising strength. Both configurations outperform the no-pore reference in toughness, whereas circular designs (I) and (II) remain below it.

## 4. Discussion

The objective of this work was to determine how the strategic placement of sub-millimeter pores may modulate the fracture resistance of ceramic material in the vicinity of fixation screws having a shaft diameter slightly larger than the pre-drilled fixation hole. It was assumed that the microstructured region of the scaffold was located at a given distance from the fixation screws. A parametric finite element study incorporating fracture propagation through the phase-field method was carried out to identify the distinct effects of pore position and pore shape on the fracture path. As a general conclusion, substantial toughening was observed, with the strongest effect associated with pore shape. In the following, a detailed discussion is presented with respect to the different features.

### 4.1. Notch Sensitivity

The response of the reference plate in Figure 2a provides a useful baseline to interpret the notch sensitivity results. Under mode I tensile loading, the plate response follows the trend predicted by Griffith’s law, with the analytical solution lying below the numerical results. This discrepancy reflects the ideal brittle nature of the Griffith model, which assumes immediate fracture upon crack initiation and neglects any energy dissipation mechanisms. In contrast, the numerical simulations allow for a finite amount of energy dissipation prior to complete failure, resulting in tensile strength values that remain above the analytical lower bound. The typical brittle failure process of the reference plate is further illustrated in Supplementary Video S1. Building on this reference behavior, a different notch sensitivity is observed for the circular scaffold. Notch opening in the circular scaffold is governed by the circumferential tensile stress induced by radial loading. According to thick-walled cylinder theory, the resulting circumferential stress field is non-uniform, with a magnitude that varies along the scaffold diameter, leading to a weak dependence of the maximum circumferential stress on notch length. In addition, a radial compressive stress component, acting locally parallel to the notch surface, may contribute to opposing notch opening. This is consistent with the experimentally observed fact that brittle ceramics behave very differently in compression than in tension: while a notch—acting as a pre-existing crack—typically propagates unstably under tensile loading, its destabilizing effect is mitigated under compression, where fracture is no longer governed by the rapid unstable propagation of a single crack, but by the slow extension of many cracks that coalesce into a crushed zone [17]. As a result, the circumferential strength of the circular scaffold remains nearly constant over the investigated range of notch sizes, deviating from Griffith-type behavior and indicating an enhanced defect tolerance compared to the plate. A similarly stable trend is observed for the normalized toughness (Figure 2b), which, unlike the monotonic decrease seen in the plate, remains remarkably insensitive to the notch size. Further insight into this behavior is provided by the stress–strain response shown in Figure 2c. The occurrence of comparable peak radial stress levels for all notch sizes indicates that fracture initiation takes place under similar global loading conditions characterized by a persistent radial compressive stress. Moreover, the absence of an abrupt post-peak stress drop and the presence of a finite transition regime demonstrate that crack initiation does not immediately lead to unstable fracture. Instead, the structure maintains a residual load-carrying capacity after crack initiation, consistent with a stabilizing stress state that delays damage evolution. This progressive damage accumulation is clearly visible in Supplementary Video S2. Overall, these results indicate that, in the circular scaffold, increasing notch size does not directly translate into a reduction of notch-opening strength, as the fracture process is strongly influenced by the combined action of circumferential tension and radial compression. This mixed stress state fundamentally alters the fracture response compared to the plate scaffold and explains the observed weak notch sensitivity.

### 4.2. Pore Position

#### 4.2.1. Radial Notch-Pore Distance

To assess the effect of pore position on fracture behavior, the notch size was fixed at a/W=0.10 for all simulations involving pores. This value was chosen to ensure that fracture consistently initiated at the notch, since smaller notches would not sufficiently concentrate stress and could cause fracture to start at the pore instead. When a pore is introduced close to the notch (i.e., $d_{r}=2R_{p}$), the overall stress levels decrease with respect to the configuration without a pore: the first peak lowers as crack initiation becomes easier, while the second peak rises sharply, indicating a significant amount of energy is required to reactivate the crack beyond the pore (Video S3). This behavior reflects the dual role of pores in brittle matrices. On one hand, the pore acts as a geometric defect that reduces the local load-bearing capacity and promotes stress concentration, leading to a decrease in strength. On the other hand, once the crack reaches the pore, a blunting effect occurs: the crack tip is arrested at the pore boundary and the stress field is redistributed over the curved pore surface. This redistribution increases the energy required for crack re-initiation, which explains the observed rise in the second peak. This mechanism is consistent with the analytical framework proposed by Leguillon and Piat [48], who showed through comparison with experimental data on porous ceramics that pores above 0.1 mm can produce toughness enhancement through crack-tip blunting. For larger distances (i.e., $d_{r}=3R_{p}$) the first peak increases approaching or even exceeding the no-pore case, suggesting greater energy is stored before failure. At the same time, the second peak is reduced, implying a less critical role of the pore in resisting crack advancement (Video S4). This behavior is consistent with the work of Rezanezhad et al. [49], who validated their XFEM approach against experimental fracture tests on specimens with predefined pore-crack configurations, and then numerically showed that notch–pore interaction is governed by their relative position within the local stress field, with increasing pore-crack distance leading to a less detrimental effect of the pore on strength. Although conducted on granite (E=70.6 GPa, $G_{c}=38.5$ J/m^2^) rather than HA (E=100 GPa, $G_{c}=10$ J/m^2^), the same trend is observed in the present study, suggesting that this mechanism is governed by the geometry of the pore-crack interaction rather than the specific material properties. These findings reflect a strong interplay between notch and pore, where the spacing modulates both initiation and propagation mechanisms. Based on these observations, the configuration with $d_{r}=3R_{p}$ was selected for the subsequent analyses, as it exhibited the highest strength among the tested cases.

#### 4.2.2. Circumferential Notch-Pore Distance

The results in Figure 4 showed that the circumferential distance of the center of the first pore with respect to the notch $d_{c}$ has a high impact on both crack path and energy dissipation. While strength progressively decreases with increasing $d_{c}$, toughness exhibits a non-monotonic trend, with a local maximum around $d_{c}=0.5R_{p}$. At this value, the crack is forced to deviate slightly before reorienting radially, leading to a modest crack deflection that increases energy absorption without compromising structural strength. As a result, this configuration provides the best strength–toughness balance among the other designs and most closely approaches the no-pore reference. This behavior contrasts with higher $d_{c}$ values (i.e., $d_{c}\geq 1.5R_{p}$), where the crack follows a longer, curved path and experiences higher energy dissipation but at the cost of strength. This behavior reflects the modulation of the notch-induced stress field by the pore’s angular position, which governs the intensity of the crack–pore interaction and the subsequent fracture deflection. This sensitivity to pore angular position is consistent with findings in brittle fracture of rocks, where Rezanezhad et al. [49] and Zhang and Shen [50] numerically showed that the angular position of the pore relative to the crack tip governs the deflection angle and the intensity of crack–pore interaction. Complementary experimental evidence on brittle materials with voids was provided by Misseroni et al. [51], who demonstrated that crack trajectories are deflected by the presence of cavities, with the deflection being directly linked to an increase in toughness. Based on this, $d_{c}=0.5R_{p}$ is selected as a reference case for subsequent analyses involving multiple pores, as it maximizes the beneficial effects of the pore without introducing excessive weakening.

#### 4.2.3. Circumferential Inter-Pore Distance

In the three-pore configuration, the parameter $d_{c}$ defined as the circumferential distance between the intermediate pore and the two other ones, plays a key role in modulating the fracture behavior. The configuration with $d_{c}=2.5R_{p}$ yielded the highest toughness among the tested geometries, but also resulted in a radially aligned crack path, reducing the influence of the central pore in guiding fracture propagation. In contrast, the configuration with $d_{c}=2R_{p}$ offered a favorable trade-off between crack deflection and mechanical performance. Although it led to slightly lower toughness, it consistently promoted a circumferential crack deviation toward the central pore (Video S6). This highlights the role of crack-path tortuosity as a fundamental dissipative mechanism, consistently observed in both experimental [51] and numerical studies [52] on brittle materials with voids. By leveraging this behavior, we achieved geometric control of the crack path, enabling enhanced energy dissipation through sequential pore engagement. In light of these reasons, $d_{c}=2R_{p}$ was selected for subsequent analyses, as it preserved controlled crack deflection while offering an increase in toughness of approximately 20% compared to the aligned configuration ($d_{c}=0R_{p}$, Video S5). However, this benefit comes at the expense of a reduced load-bearing capacity, with strength decreasing by 7%. This pattern is consistent with the well-known inverse relationship between strength and toughness in brittle ceramics [18,19,53]. However, despite the beneficial effects of pore-induced crack deflection, none of the circular pore configurations exceeds the toughness of the no-pore reference. This may be due to the interplay between stress-concentration and crack path on the overall fracture.

Interestingly, the fracture behavior observed when a single pore is circumferentially offset from the notch (as discussed in Section 4.2.1) is echoed in the configuration with $d_{c}=2R_{p}$, where a second pore is introduced along the same radial level. In both cases, the presence of a pore laterally displaced from the main fracture path promotes crack deflection, leading to reduced strength but increased toughness. This similarity suggests that it is not the specific identity or location of the pore that governs the mechanical response, but rather its angular displacement relative to the crack propagation direction. The resulting strength–toughness trade-off induced by circumferential pore deflection therefore indicates a broader, location-independent mechanism of crack redirection and energy redistribution.

### 4.3. Pore Shape

Within the framework of composites mechanics it is well established that pores, considered as a second phase, can be a primary toughening mechanism in brittle matrices [33,34,37,38,39,40,54]. Our simulations, building on this general concept, further highlight how pore shape and location can be tuned according to the specific scaffold configuration and biomedical application, to improve strength and toughness. Replacing circular pores with circumferential elongated ones of equal area, while maintaining the same spatial arrangement, produces a marked increase in toughness. A circumferentially elongated pore, oriented with its major axis perpendicular to the crack propagation direction, presents a lower effective stress concentration relative to a circular pore under the same loading, thereby promoting a more uniform stress distribution along its perimeter. This geometric effect is consistently associated with reduced crack driving forces and enhanced resistance to crack advance, as evidenced by numerical studies highlighting stress redistribution and crack deflection/trapping mechanisms [48,55,56] and by experimental observations reporting improved fracture resistance in porous materials with elongated pores [52]. This effect is confirmed by comparing circular and elongated pore configurations: (I) versus (III), both radially aligned, and (II) versus (IV), both with a circumferential offset. In both cases, the elongated pore configurations achieve higher toughness, with configuration (III) showing a ∼42% improvement over (I) (Video S7). This gain can be attributed to the local curvature of the surface from which the crack resumes propagation upon exiting the pore: while a circular pore presents a surface characterized by its radius of curvature, an elongated pore presents a comparatively flatter surface, from which crack re-initiation requires more energy. This reduces the local stress intensification and promotes a more dissipative failure process (IV versus II, Video S8). This synergy provides a performance boost: configuration (IV) shifts towards the upper-right quadrant of the strength–toughness diagram, achieving an increase in both fracture toughness and ultimate breakup value compared to configuration (II). Crucially, while typical composite architectures often incur strength penalties to gain toughness [18,19], this pore morphology enables a concurrent improvement of both properties. A further indication of the effectiveness of this pore morphology emerges from the comparison with the no-pore reference. While circular pore configurations fail to outperform the fully dense design in terms of toughness, elongated pores overcome this limitation by reducing local stress intensification, resulting in a toughness increase of approximately 30%. This improvement is achieved at the cost of only a modest strength reduction (3–6%), representing a favorable mechanical trade-off.

### 4.4. Limitations

This study presents some limitations: the analysis was performed in a simplified bi-dimensional geometry, whereas a real three-dimensional scenario may introduce additional mechanisms of interaction between cracks and porosity. Another source of uncertainty is associated with the $G_{c}$ parameter, which was estimated from existing literature data rather than measured through ad hoc experimental tests on 3D-printed hydroxyapatite. The results reported in the Supplementary Material (Figure S1) show how $G_{c}$ effects the absolute magnitude of the mechanical response, while no changes in crack trajectories were observed. These findings confirm that the comparative conclusions drawn in this study are robust with respect to uncertainty in this parameter, which motivated the choice of reporting numerical results in normalized form rather than as absolute physical values. Finally, the mesh resolution was selected according to a geometric criterion, ensuring that the characteristic element size was always smaller than 10% of the pore diameter; a further systematic mesh-convergence analysis could be performed to further improve the quantitative robustness of the analysis.

## 5. Conclusions

This study has demonstrated that the strategic placement of micro-pores in the vicinity of screw insertion sites can significantly enhance the fracture resistance of hydroxyapatite ceramic scaffolds. Through finite element simulations based on a phase-field approach for brittle fracture, the key geometrical parameters governing crack propagation paths and the strength–toughness balance were identified. First, pore shape emerges as a key design parameter. Circumferentially elongated pores, oriented perpendicular to the expected crack propagation direction, reduce stress concentration and enhance toughness beyond that of the dense peri-screw region. In contrast, circular pores do not provide toughness improvement over the same region. A moderate circumferential offset of the pores relative to the crack initiation site enhances toughness without severely compromising strength. Finally, a circular scaffold geometry confers an inherent defect tolerance compared to plate-like geometries.

These results are obtained within a two-dimensional modeling framework, which allows for a systematic investigation of in-plane crack–pore interactions. However, this representation necessarily idealizes the three-dimensional nature of real scaffolds. In particular, out-of-plane effects are not explicitly considered. In addition, the critical energy release rate ($G_{c}$) was estimated from literature rather than derived from specific experimental measurements on 3D-printed hydroxyapatite. Within this framework, the identified mechanisms provide useful guidelines for scaffold design, while also motivating future developments toward fully three-dimensional models, heterogeneous variations of pore positions and shapes, experimental calibration of material fracture parameters, and experimental validation of the identified toughening configurations on 3D-printed hydroxyapatite specimens, to further enhance the robustness of the analysis.

This work represents a first study in this direction; future developments involving more extensive parametric studies or optimization frameworks could further refine the identified design guidelines toward a fully optimized placement and shape of pores in the vicinity of the screw insertion site.

## Figures and Tables

**Table 1:** Summary of tested configurations and simulation parameters.

Parameter	Specification
Geometry	
Type	Circular/Plate (reference)
Circular width (*W*)	8 mm
Central hole radius ($R_{s}$)	1 mm
Plate width (*W*)	8 mm
Plate height (*H*)	14 mm
Out-of-plane thickness (*T*)	1 mm
Material properties	
Young’s modulus (*E*)	100 GPa
Poisson’s ratio (ν)	0.3
Tensile strength	100 MPa
Compressive strength	1000 MPa
Energy release rate ($G_{c}$)	10 J/m^2^ (reference)
	0.5, 1, 2, 5, 20 J/m^2^(sensitivity analysis)
Notch sensitivity (Circular: FE; Plate: FE + Analytical)	
Notch size (a/W)	[0.05, 0.10, 0.15, 0.20, 0.50]
Pore shape	
Geometry	Circular/Elongated (equal area)
Pore radius ($R_{p}$)	0.5 mm
Pore position	
Radial notch-pore distance ($d_{r}/R_{p}$)	[2, 2.5, 3, 3.5]
Circumferential notch-pore distance ($d_{c}/R_{p}$)	[0, 0.5, 1, 1.5, 2, 2.5, 3]
Circumferential inter-pore distance ($d_{c}/R_{p}$)	[0, 0.5, 1, 1.5, 2, 2.5]
Load	
Radial displacement $U_{r}$ (circular)	0.004 mm
Axial displacement $U_{a}$ (plate)	0.004 mm

## Data Availability

Data is contained within the article or Supplementary Material.

## Supplementary Materials

The following supporting information can be downloaded at: https://www.mdpi.com/article/10.3390/biomimetics11070479/s1. **Figure S1:** Stress at crack initiation as a function of $G_{c}$. **Video S1:** Fracture behavior of the plate scaffold with a notch a/W=0.10. **Video S2:** Fracture behavior of the circular scaffold with a notch a/W=0.10. **Video S3:** Fracture propagation in the presence of a pore located at $d_{r}=2R_{p}$. **Video S4:** Fracture propagation in the presence of a pore located at $d_{r}=3R_{p}$. **Video S5:** Fracture propagation in the presence of circular pores with $d_{c}=0R_{p}$. **Video S6:** Fracture propagation in the presence of circular pores with $d_{c}=2R_{p}$. **Video S7:** Fracture propagation in the presence of circumferentially elongated pores with $d_{c}=0R_{p}$. **Video S8:** Fracture propagation in the presence of circumferentially elongated pores with $d_{c}=2R_{p}$.
