# Supplementary material for: Strategic Placement of Pores to Modulate Toughness in Hydroxyapatite Bone Scaffolds Fixation
Source: Biomimetics (Basel). 2026 Jul 9;11(7):479. doi: 10.3390/biomimetics11070479 (PMC13406196; doi:10.3390/biomimetics11070479)
Supplement: Supplementary file 1 [file biomimetics-11-00479-s001.zip › Supplementary_Materials_HS.pptx]

## Slide 1
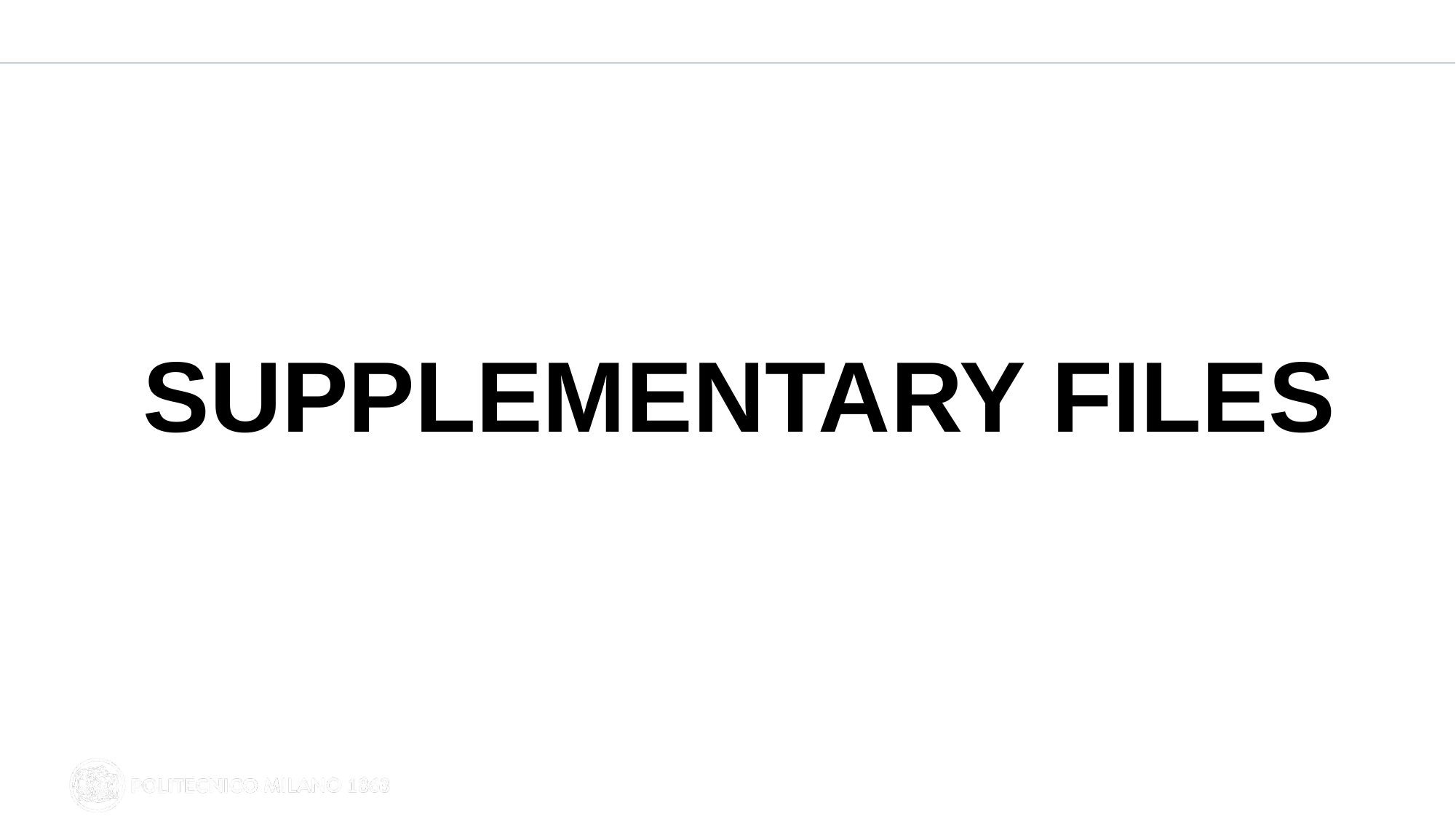

SUPPLEMENTARY FILES

## Slide 2
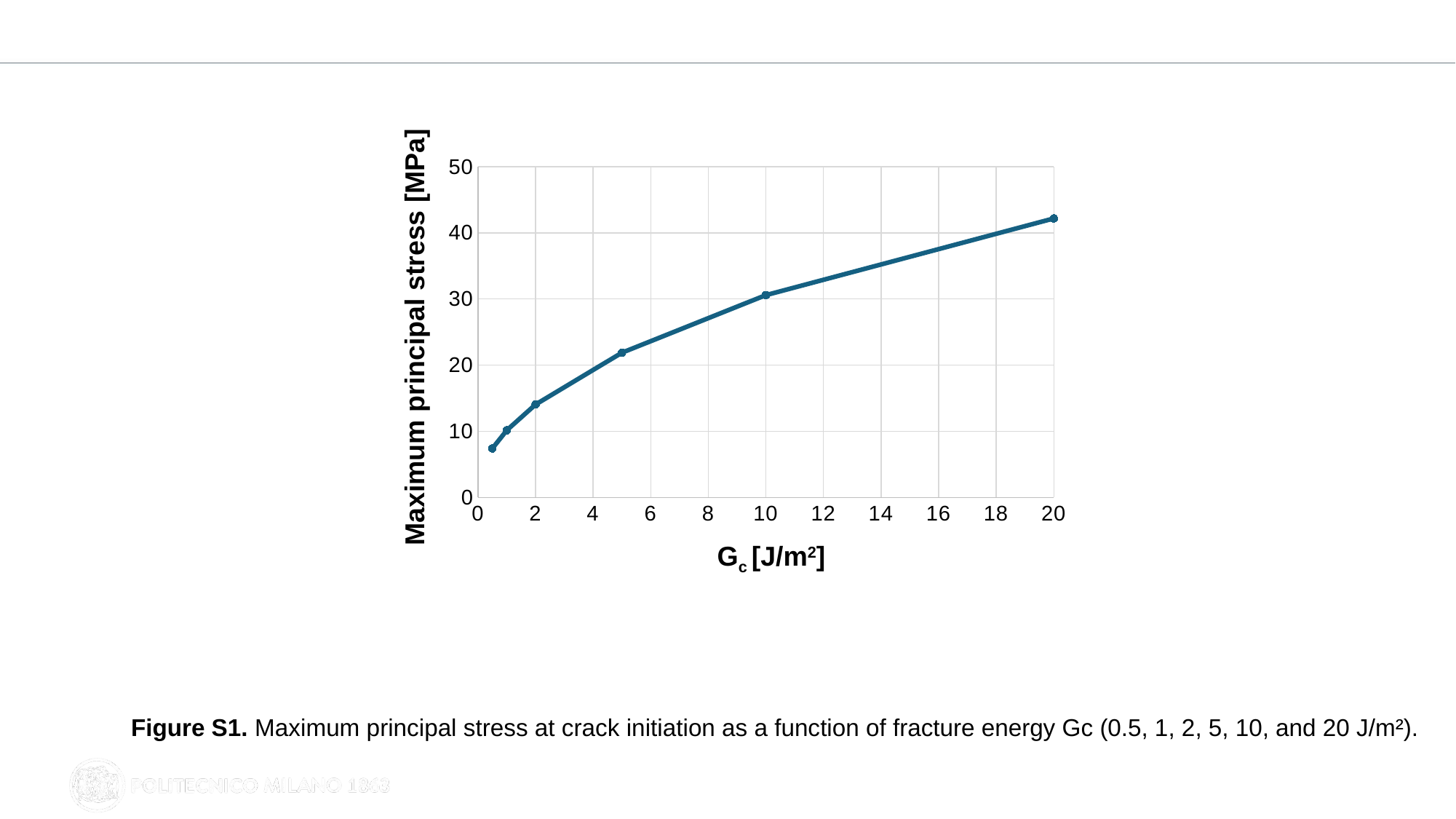

### Chart
| Category | |
|---|---|Maximum principal stress [MPa]
 Gc [J/m2]
Figure S1. Maximum principal stress at crack initiation as a function of fracture energy Gc (0.5, 1, 2, 5, 10, and 20 J/m²).

## Slide 3
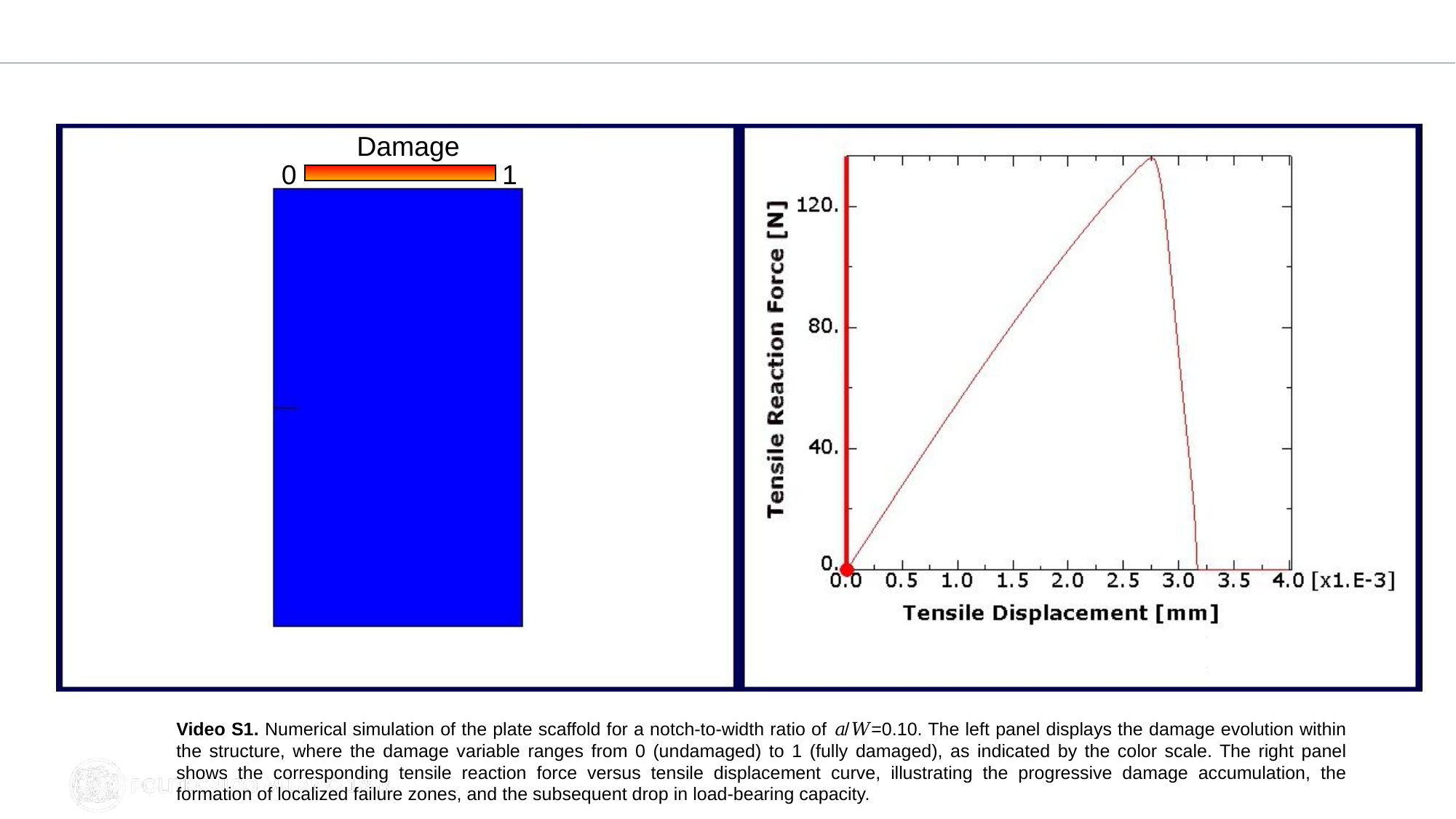

0 1
Damage
Video S1. Numerical simulation of the plate scaffold for a notch-to-width ratio of 𝑎/𝑊=0.10. The left panel displays the damage evolution within the structure, where the damage variable ranges from 0 (undamaged) to 1 (fully damaged), as indicated by the color scale. The right panel shows the corresponding tensile reaction force versus tensile displacement curve, illustrating the progressive damage accumulation, the formation of localized failure zones, and the subsequent drop in load-bearing capacity.

## Slide 4
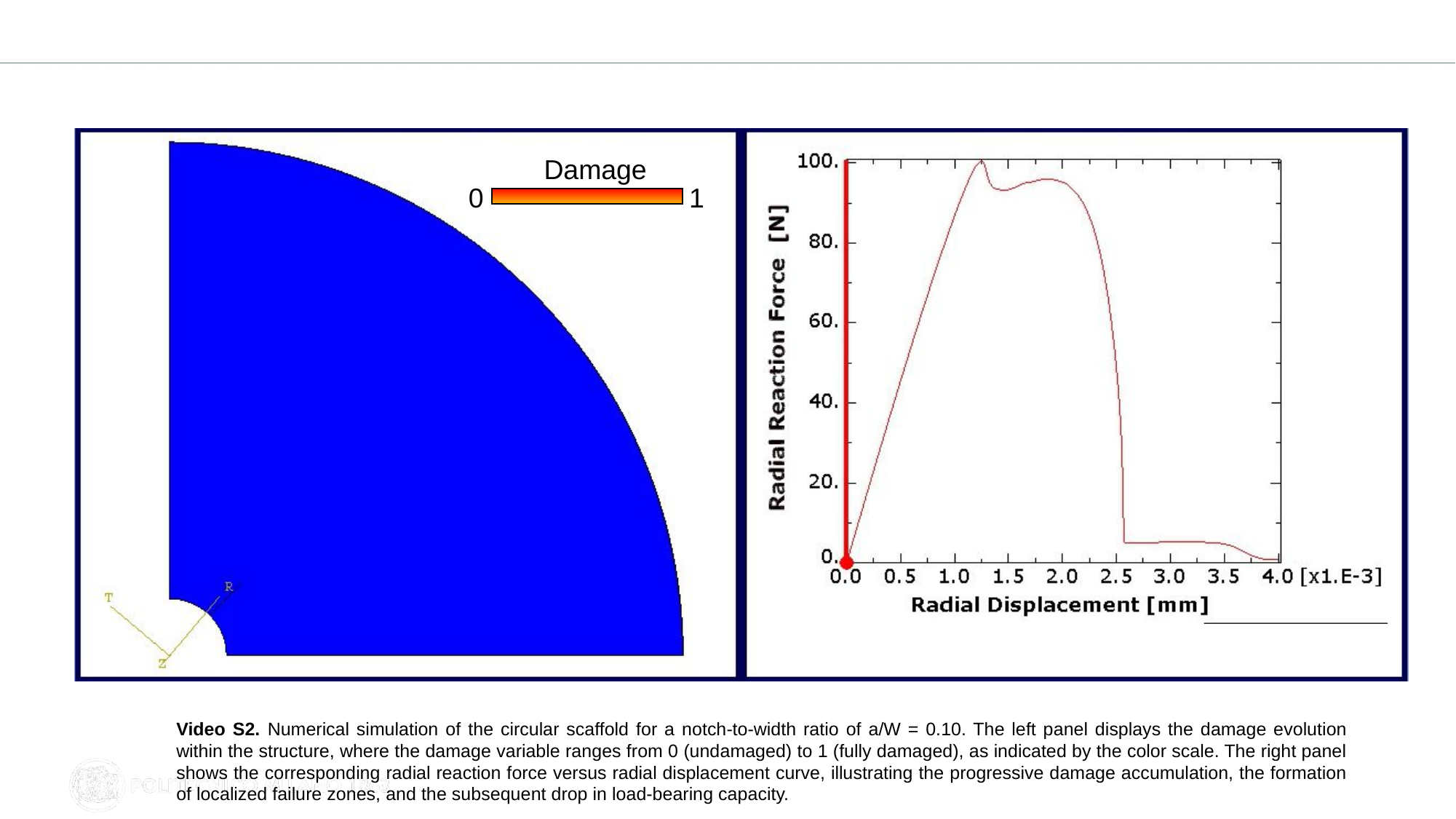

0 1
Damage
Video S2. Numerical simulation of the circular scaffold for a notch-to-width ratio of a/W = 0.10. The left panel displays the damage evolution within the structure, where the damage variable ranges from 0 (undamaged) to 1 (fully damaged), as indicated by the color scale. The right panel shows the corresponding radial reaction force versus radial displacement curve, illustrating the progressive damage accumulation, the formation of localized failure zones, and the subsequent drop in load-bearing capacity.

## Slide 5
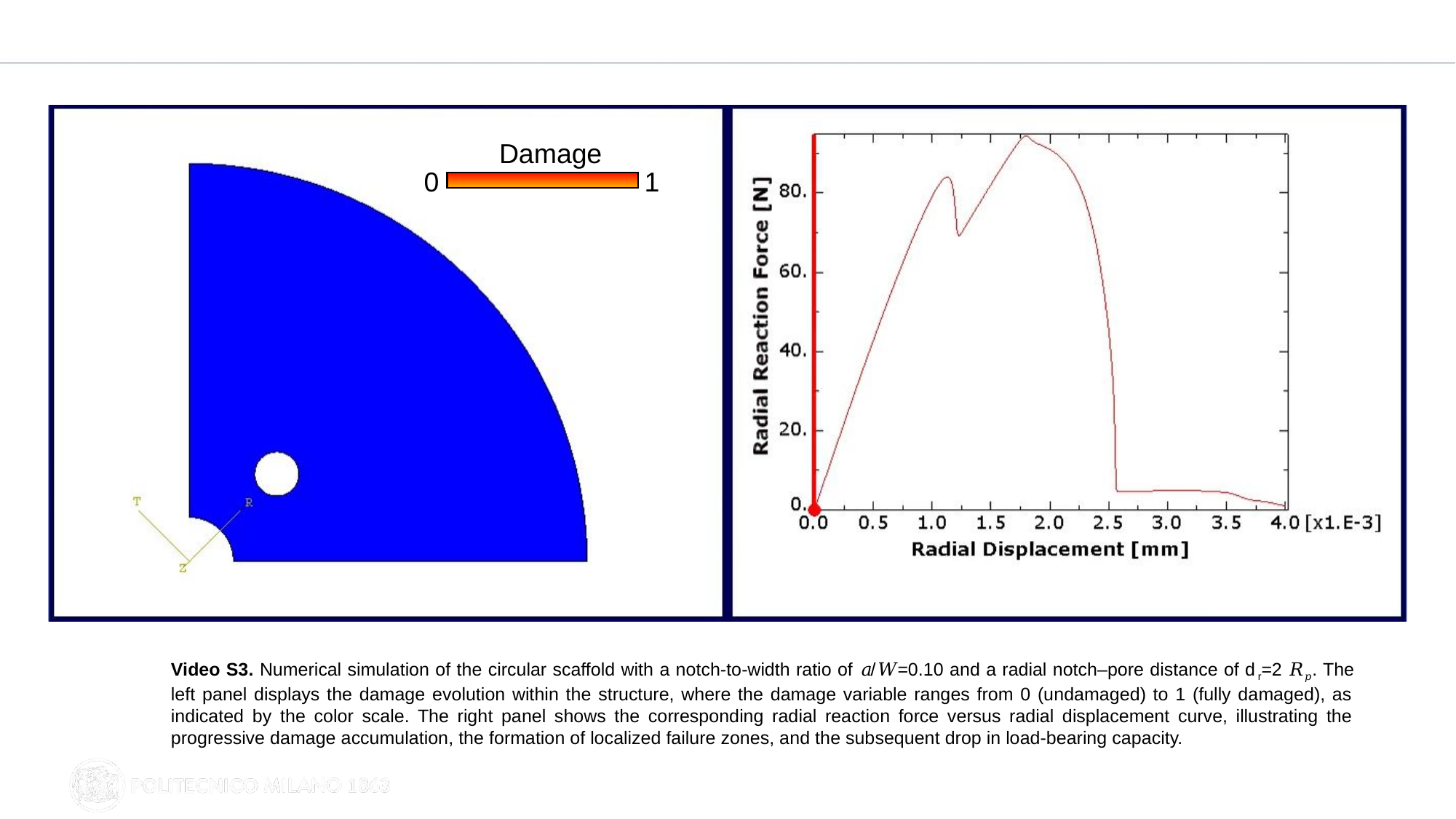

0 1
Damage
Video S3. Numerical simulation of the circular scaffold with a notch-to-width ratio of 𝑎/𝑊=0.10 and a radial notch–pore distance of dr=2 𝑅𝑝​. The left panel displays the damage evolution within the structure, where the damage variable ranges from 0 (undamaged) to 1 (fully damaged), as indicated by the color scale. The right panel shows the corresponding radial reaction force versus radial displacement curve, illustrating the progressive damage accumulation, the formation of localized failure zones, and the subsequent drop in load-bearing capacity.

## Slide 6
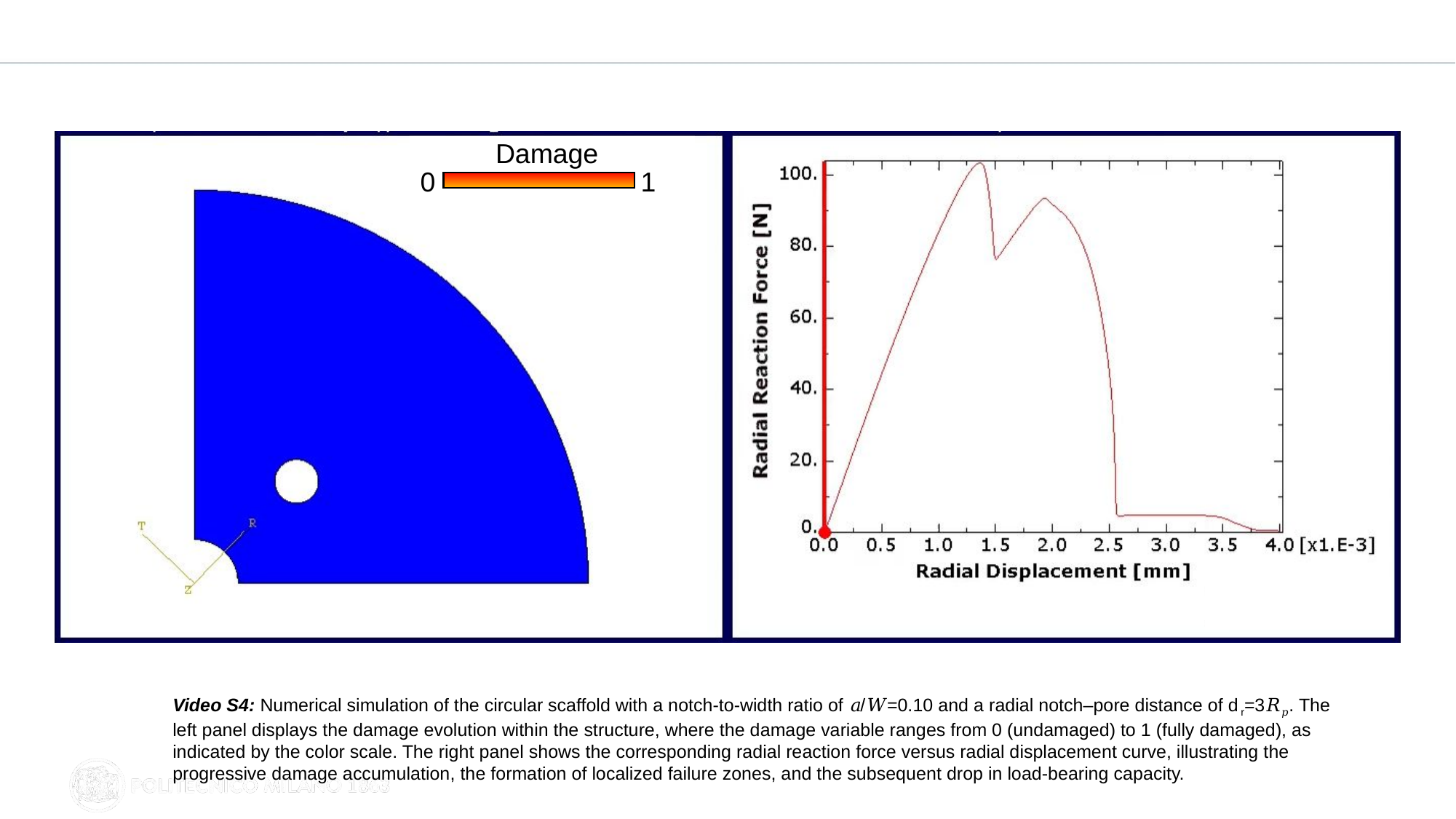

0 1
Damage
Video S4: Numerical simulation of the circular scaffold with a notch-to-width ratio of 𝑎/𝑊=0.10 and a radial notch–pore distance of dr=3𝑅𝑝​. The left panel displays the damage evolution within the structure, where the damage variable ranges from 0 (undamaged) to 1 (fully damaged), as indicated by the color scale. The right panel shows the corresponding radial reaction force versus radial displacement curve, illustrating the progressive damage accumulation, the formation of localized failure zones, and the subsequent drop in load-bearing capacity.

## Slide 7
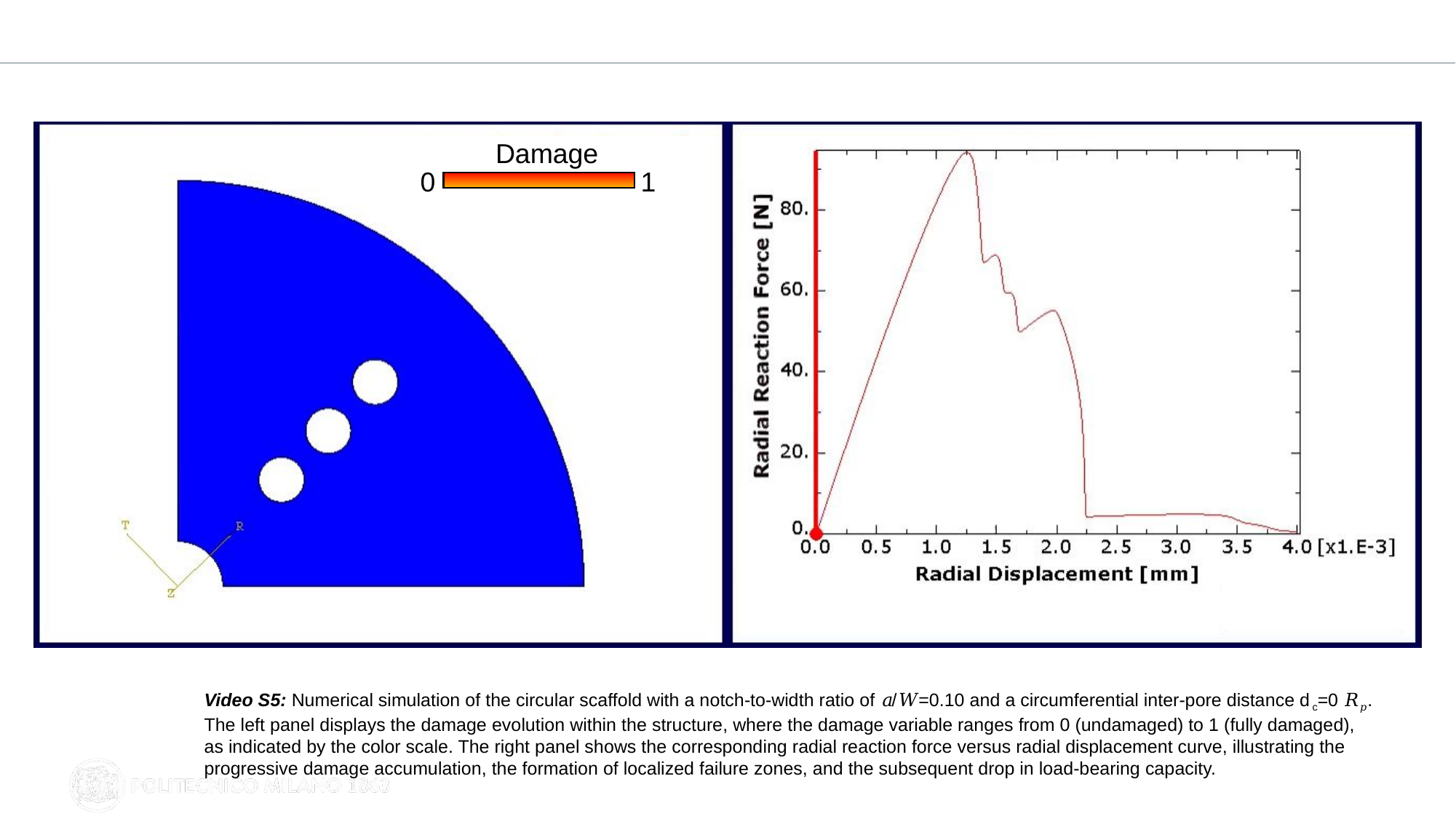

0 1
Damage
Video S5: Numerical simulation of the circular scaffold with a notch-to-width ratio of 𝑎/𝑊=0.10 and a circumferential inter-pore distance dc=0 𝑅𝑝​. The left panel displays the damage evolution within the structure, where the damage variable ranges from 0 (undamaged) to 1 (fully damaged), as indicated by the color scale. The right panel shows the corresponding radial reaction force versus radial displacement curve, illustrating the progressive damage accumulation, the formation of localized failure zones, and the subsequent drop in load-bearing capacity.

## Slide 8
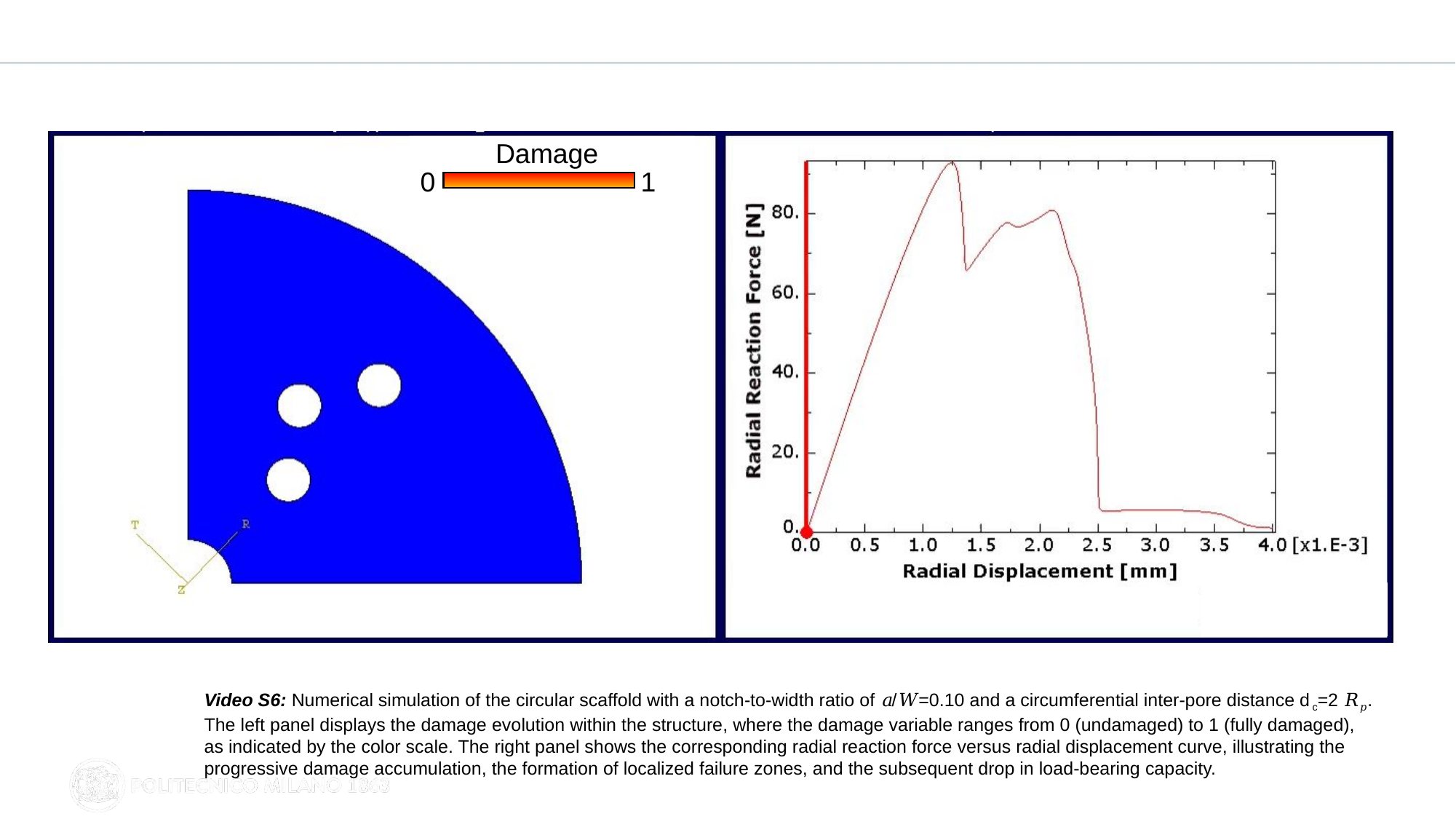

0 1
Damage
Video S6: Numerical simulation of the circular scaffold with a notch-to-width ratio of 𝑎/𝑊=0.10 and a circumferential inter-pore distance dc=2 𝑅𝑝​. The left panel displays the damage evolution within the structure, where the damage variable ranges from 0 (undamaged) to 1 (fully damaged), as indicated by the color scale. The right panel shows the corresponding radial reaction force versus radial displacement curve, illustrating the progressive damage accumulation, the formation of localized failure zones, and the subsequent drop in load-bearing capacity.

## Slide 9
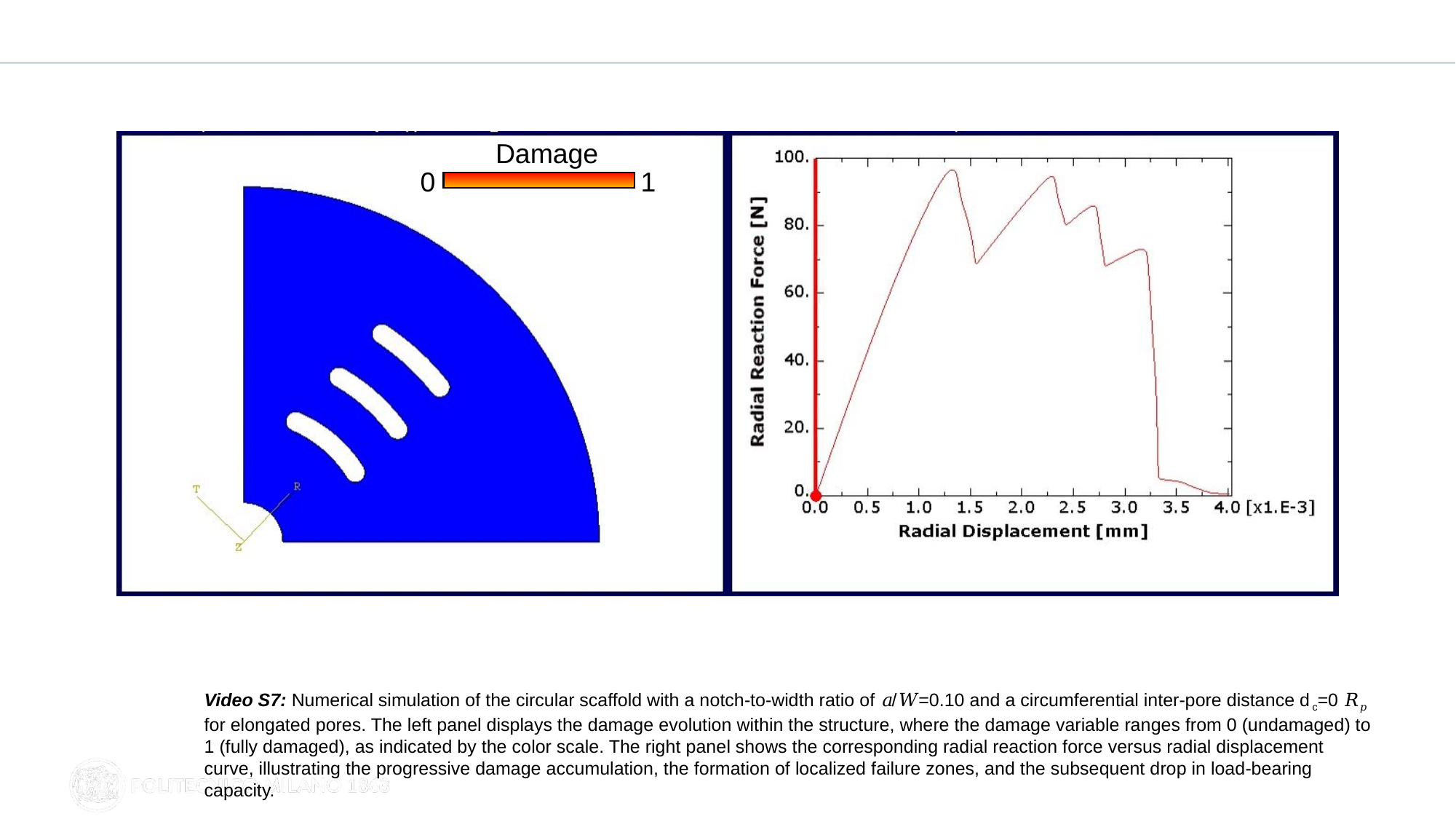

0 1
Damage
Video S7: Numerical simulation of the circular scaffold with a notch-to-width ratio of 𝑎/𝑊=0.10 and a circumferential inter-pore distance dc=0 𝑅𝑝​ for elongated pores. The left panel displays the damage evolution within the structure, where the damage variable ranges from 0 (undamaged) to 1 (fully damaged), as indicated by the color scale. The right panel shows the corresponding radial reaction force versus radial displacement curve, illustrating the progressive damage accumulation, the formation of localized failure zones, and the subsequent drop in load-bearing capacity.

## Slide 10
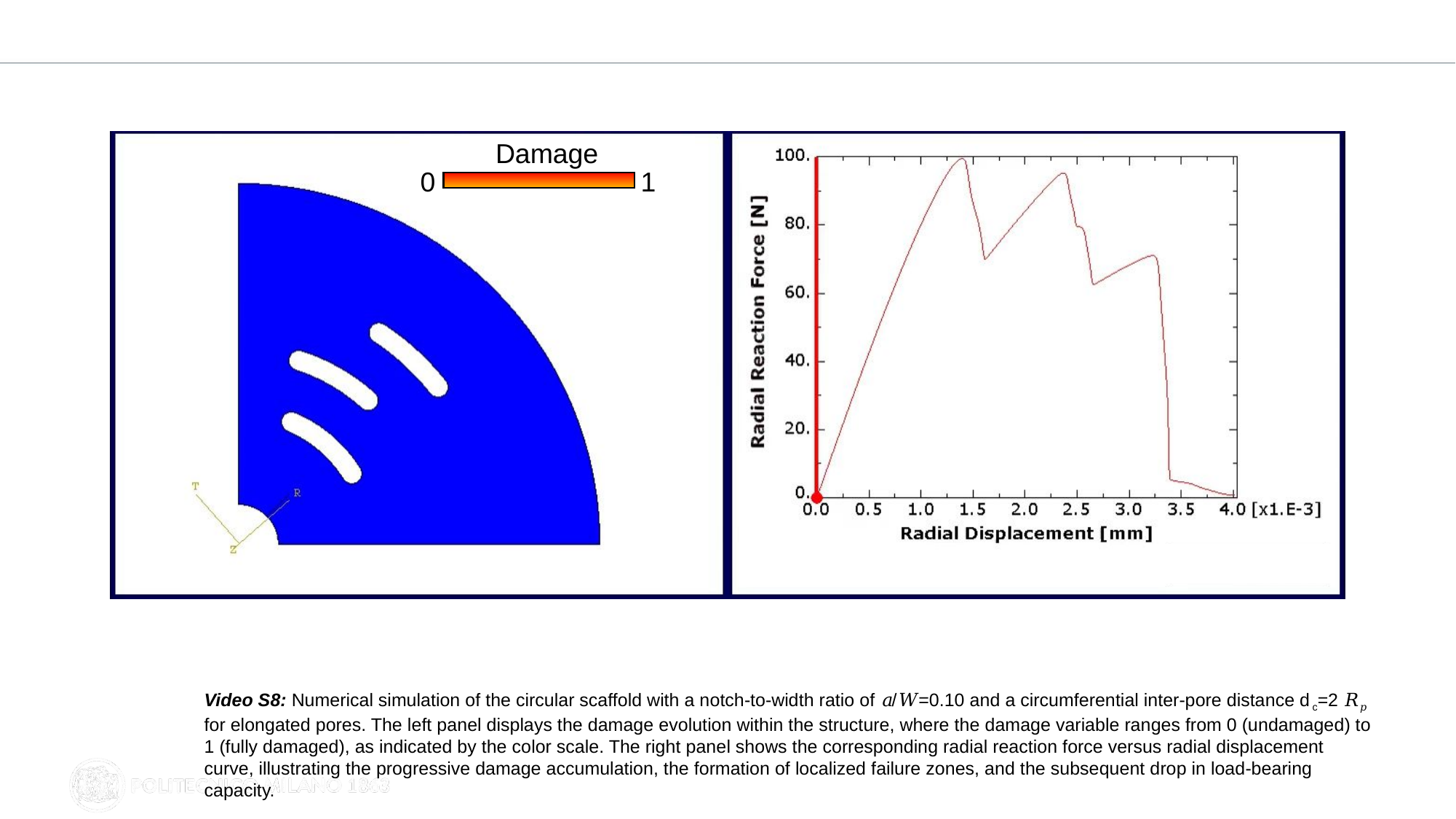

0 1
Damage
Video S8: Numerical simulation of the circular scaffold with a notch-to-width ratio of 𝑎/𝑊=0.10 and a circumferential inter-pore distance dc=2 𝑅𝑝​ for elongated pores. The left panel displays the damage evolution within the structure, where the damage variable ranges from 0 (undamaged) to 1 (fully damaged), as indicated by the color scale. The right panel shows the corresponding radial reaction force versus radial displacement curve, illustrating the progressive damage accumulation, the formation of localized failure zones, and the subsequent drop in load-bearing capacity.
